# The relationship between workers’ self-reported changes in health and their attitudes towards a workplace intervention: lessons from smoke-free legislation across the UK hospitality industry

**DOI:** 10.1186/1471-2458-12-324

**Published:** 2012-05-02

**Authors:** Laura MacCalman, Sean Semple, Karen S Galea, Martie Van Tongeren, Scott Dempsey, Shona Hilton, Ivan Gee, Jon G Ayres

**Affiliations:** 1 Scottish Centre for Indoor Air, Institute of Occupational Medicine, Edinburgh, UK; 2 Scottish Centre for Indoor Air, University of Aberdeen, Aberdeen, UK; 3 MRC/CSO Social and Public Health Sciences Unit, Glasgow, UK; 4 Centre for Public Health, Liverpool John Moore’s University, Liverpool, UK; 5 Institute of Occupational and Environmental Medicine, University of Birmingham, Birmingham, UK

**Keywords:** ‘Self-Reported Health’, Attitudes, ‘Workplace Intervention’, ‘Public Health Intervention’

## Abstract

**Background:**

The evaluation of smoke-free legislation (SFL) in the UK examined the impacts on exposure to second-hand smoke, workers’ attitudes and changes in respiratory health. Studies that investigate changes in the health of groups of people often use self-reported symptoms. Due to the subjective nature it is of interest to determine whether workers’ attitudes towards the change in their working conditions may be linked to the change in health they report.

**Methods:**

Bar workers were recruited before the introduction of the SFL in Scotland and England with the aim of investigating their changes to health, attitudes and exposure as a result of the SFL. They were asked about their attitudes towards SFL and the presence of respiratory and sensory symptoms both before SFL and one year later. Here we examine the possibility of a relationship between initial attitudes and changes in reported symptoms, through the use of regression analyses.

**Results:**

There was no difference in the initial attitudes towards SFL between those working in Scotland and England. Bar workers who were educated to a higher level tended to be more positive towards SFL. Attitude towards SFL was not found to be related to change in reported symptoms for bar workers in England (Respiratory, p = 0.755; Sensory, p = 0.910). In Scotland there was suggestion of a relationship with reporting of respiratory symptoms (p = 0.042), where those who were initially more negative to SFL experienced a greater improvement in self-reported health.

**Conclusions:**

There was no evidence that workers who were more positive towards SFL reported greater improvements in respiratory and sensory symptoms. This may not be the case in all interventions and we recommend examining subjects’ attitudes towards the proposed intervention when evaluating possible health benefits using self-reported methods.

## Background

In the past 15 years many countries have attempted to limit exposure to second-hand smoke (SHS) by implementing smoke-free legislation (SFL). In fact all EU member states have legislation in place aimed at reducing the exposures to SHS but they vary widely in terms of exemptions allowed and the enforcement of the legislation within the country [1]. Scotland was the first UK country to enforce comprehensive smoke-free restrictions in all enclosed public spaces including bars, restaurants and other workplaces (March 2006) [2]. This was followed by Northern Ireland (April 2007) [3], Wales (April 2007) and England (July 2007) [4]. The Republic of Ireland had implemented their SFL before the UK (March 2004) [5].

The impact of introducing SFL in several countries has been examined and evaluated in several ways including: changes in air quality within pubs and bars [6-9], salivary cotinine levels both population wide [10] and amongst bar workers, [11-13] hospital admissions for acute coronary syndromes [14-16] and asthma [17], changes to children’s and non-smokers’ exposure to SHS [18,19] and changes in the smoking habits of smoking hospitality workers as a result of SFL [20]. Evaluation of the health benefits of the occupational group that was most highly exposed prior to legislation, workers in the hospitality trade, has tended to be examined either by self-reported changes in symptoms [12,21-23] or by self-reported symptoms with lung function tests [13,24].

It is recognised that interviewer bias can be an important weakness in many epidemiological studies [25] but relatively little literature exists on the role of public health campaigns in influencing self-reported changes to health during evaluation studies. One study in Norway has examined the possible influence of bar workers’ attitudes to SFL and how this was related to their perception of changes in patronage for their workplace [26]. This study suggests that those with negative attitudes to SFL were more likely to report a negative economic impact post-ban.

Studies of the effect of SFL in Scotland and England during 2006–7 gathered data on participants’ attitudes towards SHS and the proposed SFL, together with data on self-reported respiratory and sensory health. These data provide the opportunity to examine the possibility that initial attitudes to the intervention under study may affect the participants’ perceived change in health. If reporting bias was demonstrated and it was shown that those who were more positive towards the legislation experienced a greater improvement in self–reported symptoms post-implementation, then the validity of such studies to demonstrate acute improvements in workers’ health is weakened.

In the case of public health interventions, such as those designed to increase physical activity, change diet and reduce alcohol consumption it is possible that members of the targeted population may feel that they are being forced to comply with a policy with which they don’t agree or that they are losing the choice that they feel they should be entitled to. In these instances attitudes towards the change or intervention may play a role in the uptake and compliance and should therefore be considered in conjunction with any investigation of self-reported health and behaviour changes.

This study aimed to investigate whether changes in self-reported symptoms were related to participants’ initial attitude towards SFL and, if this was the case, to attempt to determine the nature of the relationship.

## Methods

### Study design

This study used data collected across two research projects which assessed the implementation of SFL in Scotland (Bar workers Health and Environmental Tobacco Smoke Exposure - BHETSE) and in England (Smokefree Bars 07). Both studies used similar tools to investigate changes in bar workers’ health and attitudes pre-implementation of SFL, with follow-up at approximately two months post-implementation and again a year after the baseline measurements were taken. Exposure, health and attitudes data from the Scottish BHETSE study have been previously published [11,21,27] while exposure and health data from the Smokefree Bars 07 study in England [28,29] are also available.

### Recruitment

#### Scotland

All bars listed in the Thompson Business Directory as being in designated postcode areas in three cities (Glasgow, Edinburgh and Aberdeen) and small towns in Aberdeenshire and the Borders areas of Scotland were identified (n = 861). The areas in which these bars were situated covered a broad range of socio-economic areas and rural/urban locations. From this list of bars a random sample were selected (n = 159), contacted by telephone and asked to take part in the study. All bar managers who expressed an interest in taking part were then sent letters and other materials describing the study to distribute to their staff. After permission was granted by the bar manger, visits were carried out at pre-arranged times to maximise recruitment of bar workers. Of the 159 bars contacted 72 (45%) agreed to participate. Convenience sampling carried out between 7th January and 25th March 2006 (phase 1) resulted in the recruitment of 371 bar workers, aged over 18 years old.

#### England

The recruitment of bar workers in England followed the same methods as in Scotland. Again all bars identified from the Thompson Business Directory within designated postcode areas in two urban areas of England (central London and Liverpool) and two rural areas (Northumbria and Cumbria) were initially selected for the study. At a later stage in phase 1 (P1) the city of Newcastle-upon-Tyne was added to the study in order to increase the number of participants. A random selection of 253 bars were contacted and of these 46 (18%) agreed to participate. Again convenience sampling was carried out when visiting these bars which resulted in the recruitment of 178 bar workers.

Further information on the recruitment can be found in Hilton *et al*. [27] and Semple *et al*. [28].

### Data collection

At each visit bar workers were asked to complete a questionnaire which enquired about their attitudes towards SFL, their self-reported respiratory symptoms (shortness of breath, wheezing, phlegm production, morning cough and other cough) and sensory symptoms (runny nose, red itchy eyes and sore scratchy throat), estimated duration of exposure to SHS and demographic information such as smoking status, age and sex. The same questionnaire was used for both the Scottish and English studies and the questions asked at each phase differed only by the tense of some of the questions (pre vs. post). The survey items were adapted from questions used in the All Ireland Study of Bar Workers’ Respiratory Health [13]. The questions relating to attitudes and symptoms are listed in the relevant results tables.

The initial sampling was carried out in the months preceding the introduction of the SFL (late winter/early spring in Scotland, spring/early summer in England). Follow- up of the participants took place 2 months after the implementation of the legislation (phase 2 (P2)) and again a year after the P1 measurements were taken (phase 3 (P3)). Data collection was done by three people in Scotland (one for each city), while in England five researchers carried out the data collection (one of whom had previously collected data for the Scotland study). The data collection was carried out by the same interviewer in each area. All of the interviewers were trained in the administration of the questionnaire and were advised to be neutral so as not to influence response.

### Statistical analysis

The data was double entered into Microsoft Excel spreadsheets and checked for discrepancies. Descriptive tables are presented as counts and percentages, as well as means and ranges, where applicable. The changes in health from P1 to P3 were examined through Mann–Whitney U and McNemar’s tests. Tests of differences in attitude between countries were carried out using Mann–Whitney U-tests, while regression analysis was used to examine any relationships between attitudes (with three levels; agree, undecided, disagree) and change in number of symptoms reported. In terms of health data we consider only P1 and P3 to take account of seasonality, therefore data from P2 is not examined here. When examining the effect of attitudes on the change in symptoms we used Question H (The smoking ban was needed to protect the health of workers) to represent the initial attitude of bar workers to SFL. No data was recorded on the deprivation of the participants themselves so we have used education as a proxy when attempting to investigate the effect of deprivation. All statistical analysis was carried out using Genstat v11 [30].

### Ethics

For the Scottish data collection, the study protocol was examined by the Grampian University Hospital Trust Ethics Committee and an Advisory Committee Group was established for reviewing and monitoring the ethical and scientific procedures. Ethical approval for the English data collection was obtained from the Research Ethics Committee of the Liverpool John Moore’s University.

## Results

A total of 549 people (371 from Scotland and 178 from England) were recruited at P1. One person was excluded from the analysis for being under 18 leaving 548 eligible bar workers who participated in the study at P1.

Table 1 shows the demographic profile of the participants, as recorded during P1, and illustrates the differences and similarities between the groups of people who took part at both P1 and P3 and those who were lost to follow-up. Those who continued to participate at P3 were, generally, not significantly different from those who dropped out after P1, with between 50 and 60% of each subgroup not taking part at P3 (e.g. 52% of males and 55% of females were lost to follow-up primarily due to the high turnover rate among students employed in bar work). One exception is that a higher proportion of bar workers in England were lost to follow-up (p < 0.001), especially those from London and Newcastle while there was a slightly lower proportion of those from Aberdeen lost to follow-up.

**Table 1:** Demographic profile of the 548 bar workers who participated in the study; those who participated at both P1 and P3 (followed-up: N = 253) and those who were lost to follow-up by P3 (N = 295)

	**Followed-up (N = 253)**		**Lost to follow-up (N = 295)**			**p-val**^**1**^
	**Mean**	**Range**	**Mean**	**Range**		
Age	31.3	(18.4, 66.7)	28.0	(18.2, 71.1)		<0.001
Hours Worked Per week	34.4	(2.0, 90.0)	31.9	(0.0, 168.0)		0.098
Years worked in this Bar	4.3	(0.0, 37.0)	2.0	(0.0, 26.0)		<0.001
Years worked in all bars	8.8	(0.0, 43.0)	6.1	(0.0, 40.0)		<0.001
	**N**	**%**	**N**	**%**	**Proportion lost to follow-up (%)**	**p-val**^**2**^
Sex						
Male	126	49.8	137	46.4	52.1	0.406
Female	127	50.2	158	53.6	55.4	
Smoking Status						
Regular	104	41.1	124	42.0	54.4	
Occasional	26	10.3	39	13.2	60.0	0.700
Ex	41	16.2	43	14.6	51.2	
Never	80	31.6	88	29.8	52.4	
*Not answered*	2	0.8	1	0.3		
Location						
Aberdeen	73	28.9	47	15.9	39.2	
Glasgow	54	21.3	67	22.7	55.4	
Edinburgh/Borders	63	24.9	66	22.4	51.2	
Liverpool	30	11.9	35	11.9	53.8	<0.001
London	9	3.6	40	13.6	81.6	
Newcastle	6	2.4	16	5.4	72.7	
Rural Cumbria and Northumbria	18	7.1	24	8.1	57.1	
Country						
England	63	24.9	115	39.0	64.6	<0.001
Scotland	190	75.1	180	61.0	48.6	
Education						
School	69	27.3	69	23.4	50.0	
Further Education College	77	30.4	92	31.2	54.4	0.232
University	102	40.3	119	40.3	53.8	
Postgraduate	5	2.0	15	5.1	75.0	
Ethnicity						
Asian	0	0.0	1	0.3	100.0	
Black	2	0.8	1	0.3	33.3	
Mixed	2	0.8	9	3.1	81.8	0.217
Other	0	0.0	1	0.3	100.0	
White	249	98.4	282	95.9	53.1	
*Not answered*			1			

The answers shown in the table are as given at P1.

^1^ P-value for the 2 sample test of equal means.

^2^ P-value for the chi-square test of equal proportions.

The questionnaire included a number of questions about bar workers’ attitudes towards SFL. Table 2 shows the answers given by all 548 people seen at P1, subdivided by country. The attitudes to the legislation were mixed with bar workers being more negative when asked questions what could be thought of as being financial (A, C and D). Bar workers felt that the legislation would make bars more comfortable (75% agreed), encourage smokers to quit (69%) and overall they agreed with the SFL (67%). The bar workers felt strongly that SFL was needed to protect the health of bar workers (81%). There was no significant difference in the initial attitudes of Scotland-based bar workers as compared to England-based bar workers, with the exception of borderline differences in the attitudes to questions A (The ban on smoking will have a negative impact on business) and D (The smoking ban will make smokers smoke more at home). As a marker for deprivation we also looked at initial attitudes by highest attained education (School, college, university or postgraduate). For the majority of the questions those who were educated to degree level and higher were significantly more positive towards the legislation than those who did not continue with education after school (Table 3).

**Table 2:** Initial attitudes of all bar workers seen at P1 in England (n = 178) and Scotland (n = 370)

			**Response**^**1**^**(%)**						
**Question**		**Country**	**N**	**1**	**2**	**3**	**4**	**5**	**p-val**^**2**^
A	The ban on smoking will have a negative effect on business for public bars	England	174	16	25	34	17	8	0.047
		Scotland	369	16	33	33	15	3	
B	The smoking ban is an unfair restriction on smokers	England	176	13	23	16	30	19	0.255
		Scotland	367	13	16	16	35	20	
C	Fewer people will visit public bars after the ban on smoking	England	174	14	26	26	29	5	0.986
		Scotland	367	11	30	28	25	6	
D	The smoking ban will make smokers smoke more at home	England	175	19	37	24	16	3	0.052
		Scotland	369	15	33	29	21	3	
E	The smoking ban will result in jobs being lost	England	176	9	15	32	37	8	0.203
		Scotland	367	8	18	33	36	4	
		**Country**	**N**	**5**	**4**	**3**	**2**	**1**	**p-val**
F	Smoke free public bars will make visits to them more comfortable	England	175	2	9	14	38	37	0.260
		Scotland	369	2	8	14	33	43	
G	The smoking ban will encourage smokers to quit	England	176	1	10	20	55	14	0.538
		Scotland	369	1	9	21	51	19	
H	The smoking ban is needed to protect the health of workers	England	175	2	5	9	42	42	0.342
		Scotland	369	1	7	12	41	39	
I	Do you agree with the proposed ban on smoking in public bars?	England	176	8	11	17	24	39	0.606
		Scotland	367	10	12	10	27	42	

^1^ Response: 1-Strongly Agree, 2- Agree, 3-Undecided, 4- Disagree, 5-Strongly Disagree.

^**2**^ The p-value is for the Mann–Whitney U test of whether there was a difference in the initial attitude of those in England to those in Scotland.

**Table 3:** Initial Attitudes of all bar workers seen at P1 in by highest level of education attained; School (138), college (169) and university and postgraduate (Uni) (241)

			**Response**^**1**^**(%)**						
**Question**			**N**	**1**	**2**	**3**	**4**	**5**	**p-val**
A	The ban on smoking will have a negative effect on business for public bars	School	137	18	27	34	15	5	
		College	165	20	33	31	13	2	0.04
		Uni	241	12	30	35	17	5	
B	The smoking ban is an unfair restriction on smokers	School	138	20	20	20	29	12	
		College	166	12	25	16	32	14	<0.001
		Uni	239	9	13	14	36	28	
C	Fewer people will visit public bars after the ban on smoking	School	138	20	24	27	28	2	
		College	163	13	33	29	21	3	0.001
		Uni	240	7	29	26	28	9	
D	The smoking ban will make smokers smoke more at home	School	137	16	37	27	16	4	
		College	166	20	36	23	20	1	0.062
		Uni	241	14	31	30	21	4	
E	The smoking ban will result in jobs being lost	School	137	12	21	33	30	4	
		College	165	10	21	32	30	7	<0.001
		Uni	241	5	12	33	44	5	
			**N**	**5**	**4**	**3**	**2**	**1**	**p-val**
F	Smoke free public bars will make visits to them more comfortable	School	138	2	11	14	42	30	0.045
		College	165	1	9	16	36	38	
		Uni	241	2	7	13	29	49	
G	The smoking ban will encourage smokers to quit	School	138	1	12	25	45	16	
		College	166	1	10	21	53	16	0.158
		Uni	241	1	7	17	55	19	
H	The smoking ban is needed to protect the health of workers	School	137	1	9	14	41	36	
		College	166	1	7	10	46	36	0.212
		Uni	241	1	5	10	39	45	
I	Do you agree with the proposed ban on smoking in public bars?	School	138	14	14	14	27	30	
		College	165	10	13	15	24	38	<0.001
		Uni	240	6	9	9	27	49	

The p-value is for the regression of whether there was a difference in the initial attitude over the different education levels.

^1^ Response: 1-Strongly Agree, 2- Agree, 3-Undecided, 4- Disagree, 5-Strongly Disagree.

The questionnaire also asked whether the participants experienced a number of respiratory symptoms (shortness of breath, wheezing, phlegm production, morning cough and other cough) and sensory symptoms (runny nose, red itchy eyes and sore scratchy throat). One of the questions in this section was whether they had a cold at the time of survey, which enabled identification of those for whom the reported symptoms could be attributed to a cold. Those who said they had a cold at P1 and/or P3 were removed for the analysis of reported health symptoms. This left 180 people (51 from England and 129 from Scotland) who were seen at both P1 and P3. Table 4 shows the change in reported symptoms from P1 to P3 of all of the remaining bar workers, subdivided by the country in which they worked.

**Table 4:** The change in reported symptoms from P1 to P3

	**Number (%) with symptoms**				**Change in Symptoms**^**1**^			
	**Baseline**		**Follow-up**		**No. Reduced**	**No. Increased**	**No. No Change**	**P-value**^**2**^
**England**								
**Respiratory symptoms**	N	%	N	%				
Median # of symptoms (IQR)^3^	1	(0, 3)	0	(0,1)	27	3	20	0.007
Any symptom	31	(61)	19	(37)	15	3	33	0.041
Wheezing/whistling	18	(35)	5	(10)	14	1	36	0.001
Shortness of Breath	11	(22)	6	(12)	7	2	42	0.180
Cough, morning	21	(41)	7	(14)	15	1	35	0.001
Cough, rest of day or night	15	(29)	10	(20)	7	2	42	0.180
Phlegm production	16	(31)	7	(14)	11	2	37	0.022
**Sensory symptoms**								
Median # of symptoms (IQR)	1	(0, 1)	0	(0,1)	22	10	18	0.037
Any symptom	28	(55)	16	(31)	20	8	23	0.041
Eyes, red or irritated	12	(24)	6	(12)	10	4	37	0.180
Nose, runny or sneezing	18	(35)	12	(24)	12	6	32	0.238
Throat, sore or scratchy	10	(20)	3	(6)	9	2	40	0.065
**All symptoms**								
Median # of symptoms (IQR)	2	(1, 4)	1	(0,2)	30	6	13	<0.001
Any symptom	39	(76)	25	(49)	18	4	29	0.017
**Scotland**								
**Respiratory symptoms**								
Median # of symptoms (IQR)	2	(0, 3)	1	(0,2)	49	24	54	0.015
Any symptom	86	(67)	70	(54)	23	7	99	0.042
Wheezing/whistling	42	(33)	29	(22)	21	8	100	0.024
Shortness of Breath	43	(33)	28	(22)	27	12	88	0.024
Cough, morning	39	(30)	28	(22)	21	10	98	0.071
Cough, rest of day or night	54	(42)	43	(33)	27	16	86	0.126
Phlegm production	50	(39)	36	(28)	20	6	103	0.009
**Sensory symptoms**								
Median # of symptoms (IQR)	1	(0, 2)	1	(0,2)	46	26	57	0.033
Any symptom	89	(69)	69	(53)	31	11	87	0.011
Eyes, red or irritated	43	(33)	24	(19)	25	6	98	0.001
Nose, runny or sneezing	60	(47)	61	(47)	23	24	82	1.000
Throat, sore or scratchy	54	(42)	39	(30)	31	16	82	0.040
**All symptoms**								
Median # of symptoms (IQR)	2	(1, 5)	2	(0,4)	59	31	37	0.011
Any symptom	104	(81)	87	(67)	6	23	100	0.016

The table shows only those who did not have a cold at either phase and is split into England (n = 51) and Scotland (n = 129).

^1^ Reduction – Symptoms at Baseline, none at follow-up: Increase No symptoms at baseline, symptoms at follow-up. For ‘median # of symptoms’ this is the number of people whose number of symptoms changed. For ‘any symptom’ this is the change in the number of people experiencing any symptom.

^2^ The p-value relates to the McNemar test for matched pairs, with exact probabilities based on a Binomial assumption with the exception of; Median number of symptoms which were tested using Mann–Whitney U test of equal medians.

^3^ The IQR is the inter-quartile range.

The proportion of people reporting any symptoms was significantly reduced from P1 to 3, in both England (76% vs. 49%) and Scotland (67% vs. 87%), with similar patterns being evident for both countries. However, the size of the reduction in symptom prevalence in Scotland was lower than in England. For example the proportion of bar workers in Scotland reporting wheezing reduced from 33 to 22%, while the reduction was from 35 to 10% of bar workers in England.

As symptom reporting is subjective it is of interest to investigate whether specific factors have an influence on the number of symptoms reported. We therefore considered whether people’s pre-legislation attitudes towards SFL was related to the change in the number of symptoms they reported between pre- and post-implementation.

The analyses of bar workers initial attitude to SHS exposure and SFL (based on their initial response to Question H) and symptom reporting were carried out separately by country due to the apparent differences in the change in health seen in Table 4.

**Figure 1:**
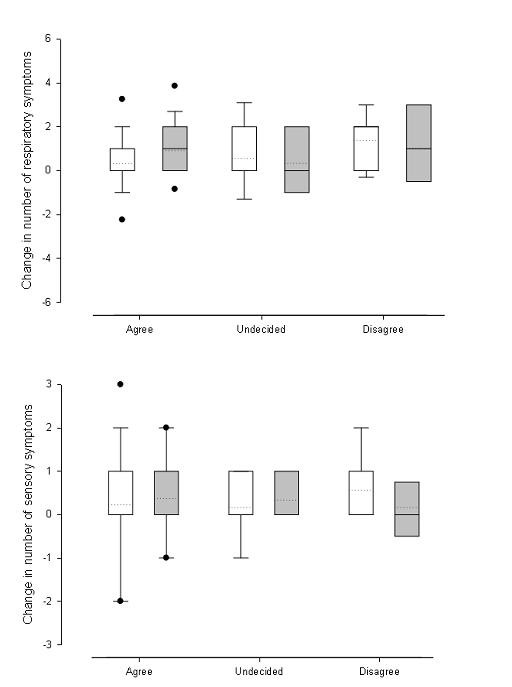
**Boxplot of the change in respiratory and sensory symptoms by initial attitude for Scotland (White) and England (Grey)**. Top: Respiratory symptoms; Bottom: Sensory symptoms. The dotted line on each box shows the mean change in number of symptoms, while the black points are outliers.

Figure 1 illustrates the change in the number of symptoms reported from P1 to P3 by attitude towards SFL (dotted line represents mean). The initial attitude to SFL did not have any effect on the change in symptoms reported by those in England (Respiratory, p = 0.755; Sensory, 0.910); it did however seem to have an effect on the average change of respiratory symptoms reported in Scotland (p = 0.042). The relationship is such that those who disagreed with the statement (i.e. were initially negative towards the need for SFL) experienced a significantly greater improvement in self-reported health than those who held positive attitudes towards the restrictions.

Investigating further it seems that there is a relationship between the initial respiratory symptoms reported by bar workers in Scotland and initial attitude (p = 0.008) but no relationship between initial attitude and health at P3 (p = 0.498). From this it seems safe to conclude that, in Scotland, the effect of initial attitude on the change in health is primarily driven by the relationship between attitude and health at P1, with the proportion of those with no symptoms being significantly higher for those who were most positive and decreasing as the scale became more negative. The results for England follow a similar pattern but the numbers in each cell of the table are so small that it is difficult to make any conclusions.

We also examined the effect that other factors, such as smoking status (model p-value ranges from 0.166 to 0.482), education (model p-value ranges from 0.491 to 0.942) and hours worked per week (model p-value ranges from 0.746 to 0.955), may have had on the change in symptoms reported and found no significant relationships, even when considered in addition to attitude.

Although question H was chosen as a surrogate for each bar worker’s overall attitude towards SHS and the need for SFL, analysis was undertaken to examine any links between attitudes expressed in the other more specific questions and changes in self-reported health. No significant relationships were found for either country between attitude and change in reported health.

## Discussion

The introduction of SFL around Europe and beyond, has enabled a novel investigation of the effects of public health interventions. The collection of contemporaneous health and attitudinal data in the evaluation of legislative changes in Scotland and England has allowed an investigation of the relationship between workers’ attitudes towards an occupational intervention and their self-reported health changes.

Initial attitude did not have an effect on the change in symptoms reported by those in England. There was, however, a relationship between the change in reported respiratory symptoms and initial attitude in Scotland. The biggest improvement in respiratory symptoms, from P1 to P3, was reported by those who were initially negative towards the SFL.

We found that the initial attitude is more likely to be associated with the symptoms reported initially, with those who were initially more positive towards the legislation being more likely to report no symptoms than those who had a negative attitude.

Our study found no evidence that those who were more in favour of the proposed intervention were more likely to report greater improvements in their health a year later, indeed the opposite appears to be true. Initially this was thought to be affected by the difference in attitudes between smokers and non-smokers with smokers being generally more negative towards the proposed change and also having poorer reported health at the study baseline thus giving them more opportunity to experience health improvement than the more positive non-smoking participants. We found though that there was no association between smoking status and change in reported health.

The same interviewers collected information on attitudes and health at all phases of the study, within each country. One of these interviewers collected data in both the Scotland and England study. The same, straightforward, questionnaire was used for both studies and all of the interviewers were trained in its administration and advised to be neutral so as not to influence response. The protocol for carrying out the interviews was designed to minimise any interviewer effect on the responses obtained from the participants.

The fact that both studies used the same methods meant that the data could be amalgamated in order to examine the effects of the SFL in the UK. It also means that the results of any analysis are directly comparable thus allowing comparison of the attitudes towards the SFL and the effect this possibly had on changes in self-reported symptoms between the two countries.

At the time of implementation there was significant press coverage and public debate surrounding the SFL .This may have, potentially, affected a number of areas of this study. The high level of agreement that SFL was needed to protect bar workers’ health may be due to the comprehensive information campaign (for example; NHS Health Scotland, The Scottish Executive and Cancer Research UK) in the months leading up to the legislation. The generally positive experience of the legislation in Ireland may also have influenced Scottish bar workers’ expectations and attitudes towards smoking restrictions. The response rate of bars that were asked to join the study could possibly have been affected by the attitude of the bar managers towards the ban, with a lower proportion of bars in England agreeing to participate (18% England; 45% Scotland), but as data were not available on the reasons for non-participation this cannot be confirmed.

The follow-up study was hampered, to some extent, by attrition of a significant proportion of participants (65% in England and 49% in Scotland). As discussed by Ayres *et al.*[21] bar staff often consist of students who only work during term-time/holidays and the populations in cities in general are typically transient. This could explain the higher rate of loss to follow-up in Edinburgh, Glasgow, London and Newcastle.

The SFL implemented in England and Scotland has been evaluated via a number of routes. From previous work on the bar workers in Scotland it was evident that smokers were more negative towards SFL, initially, than non-smokers [29] and that the attitudes of the smokers underwent a greater improvement following the implementation of the legislation. It is likely that this may be due to smokers realising that the legislation did not have such a negative impact on them and their workplaces as they feared.

The bar workers were initially quite negative regarding the financial impact of the legislation. It did, however, appear that a high proportion of bar workers felt that SFL would make the bars more comfortable to work in and be better for workers’ health. So while they felt there was the possibility that the legislation would have a negative impact on business they did see that it was needed to protect their health.

There was no real difference in initial attitudes between England and Scotland. This is perhaps surprising, due to the publicity surrounding the effects that the Scottish legislation had on health and exposure to SHS in the year following implementation (the year leading up to its introduction in England). It might have been hypothesised that the positive experience of Scotland as a result of the legislation would improve the initial attitudes of those in England when the legislation was introduced a year later, but this does not seem to have been the case. The bar workers in England were, however, more negative, initially, towards the financial aspects of the legislation than those in Scotland. This could have been impacted by reports of bars closing in Scotland where the blame was given to SFL.

The results reported here examine health symptoms reported in the run up to the introduction of SFL and again a year later. Results were collected a year later in order to take account of seasonality, which would likely have an effect on symptoms being reported, due to the weather. Those with a cold at either phase were excluded from the analysis of self-reported health symptoms to attempt to exclude those who were suffering from any or all of these symptoms due to an illness.

In both countries symptoms reduced from P1 to P3, but there was a bigger decrease in England. It is possible that there is higher chance of having cold-like symptoms in the Spring (when Scotland data was collected) than in the Summer (England data collection), although the removal of those who reported having a cold should have reduced the impact this would have had. Previous analyses also demonstrated that smokers had more symptoms than non-smokers at P1 [21,29] and so it is possible that these smokers thus had more capacity to experience health improvement than the non-smoking group.

The majority of studies investigating the effect of SFL around the world make use of self-reported health symptoms. Many of these studies also collected information on attitudes towards the legislation, but most do not report any investigation of the effect that attitudes may have on changes in self-reported health.

One study [23] found that, among hospitality workers in Sweden, there was a larger decline in the prevalence of symptoms for those who felt more positive towards the legislation. They suggested that selection bias could have contributed to this as their study sample consisted of volunteers. This study used a very different measure for the attitudes and the participants were generally more positive towards the legislation being implemented. Another study investigating the impact of SFL in Norway reported that initial attitudes towards the legislation influenced subjective reports of economic effects of the SFL [26]. This study of over 500 bar and restaurant workers found a negative pre-ban attitude towards SFL significantly increased the odds for reporting a negative economic impact post-ban.

It is clear that there are many factors which could affect the bar workers’ feelings towards SFL including their age and their own smoking habits. Their personal feelings towards SFL may have also been affected by positive and/or negative publicity concerning the health effects of SHS and SFL, as well as the possible financial effects to their workplace.

This study highlights the complex interplay of knowledge, attitudes and perceptions of changes to the working environment. Making workers more aware of the hazards associated with their working environment and of the potential benefits to their health and working life that a proposed intervention could have will all play a role in bringing about compliance with control measures.

In general, workplace interventions which are aimed at improving health and safety tend to be evaluated by looking at changes in health and behaviours using self-reported information, ideally in conjunction with some objective measure of change. Although we have reported here that the initial attitudes of workers tended not to introduce bias in the reported changes in health, it is clear that this should be considered when designing evaluation studies of complex occupational interventions.

## Conclusions

The findings here provide support to other studies that have used self-report as a method for assessing complex workplace interventions. We did not find any evidence that those with positive attitudes towards the change being proposed to their workplace were more likely to report health benefits after implementation.

However, we feel that studies investigating the impact of occupational or public health interventions should consider examining the attitudes of the participants to the intervention itself, as would be done with the characteristics of the participants. This will allow research teams to determine if attitudes may play a role in introducing bias to self-reported health changes.

## Competing interests

None of the authors have any competing interest.

## Authors’ contributions

LM carried out the statistical analysis of the data and has drafted this manuscript. SS was the principal investigator for the England bar workers study, project manager for the Scotland bar workers study and was involved in the development of the concept for this paper. KG collaborated on both the Scottish and English bar workers study, while MVT collaborated on the English bar workers study. Both were involved in the analysis and interpretation of the data presented. JA was the principal investigator for the Scotland bar workers study and involved in the design of the BHETSE study. SD, SH and IG collected all of the data analysed here and were involved in the design of the respective studies in which they were involved. All of the authors have read and commented on this and previous versions of the manuscript.

## What this paper adds

Little has been published concerning the effect of attitudes towards a public health or workplace intervention on changes in self-reported health due to said intervention. The findings reported in this paper support the use of self-report as a method of evaluating public health and workplace interventions. We suggest that future evaluation of public health and workplace interventions should consider participants' attitudes towards the intervention.

## Pre-publication history

The pre-publication history for this paper can be accessed here:

http://www.biomedcentral.com/1471-2458/12/324/prepub
